# Mechanistic Understanding of Peptide Analogues, DALDA, [Dmt^1^]DALDA, and KGOP01, Binding to the Mu Opioid Receptor

**DOI:** 10.3390/molecules25092087

**Published:** 2020-04-29

**Authors:** Maria Dumitrascuta, Marcel Bermudez, Steven Ballet, Gerhard Wolber, Mariana Spetea

**Affiliations:** 1 Department of Pharmaceutical Chemistry, Institute of Pharmacy and Center for Molecular Biosciences Innsbruck (CMBI), University of Innsbruck, Innrain 80-82, 6020 Innsbruck, Austria; maria.dumitrascuta@uibk.ac.at; 2 Institute of Pharmacy, Freie Universität Berlin, Königin-Luise-Str. 2+4, D-14195 Berlin, Germany; m.bermudez@fu-berlin.de; 3 Research Group of Organic Chemistry, Departments of Chemistry and Bioengineering Sciences, Vrije Universiteit Brussel, Pleinlaan 2, B-1050 Brussels, Belgium; Steven.Ballet@vub.be

**Keywords:** mu opioid receptor, opioid peptides and peptidomimetics, DAMGO, DALDA, [Dmt^1^]DALDA, KGOP01, binding, molecular docking, structure-activity relationships

## Abstract

The mu opioid receptor (MOR) is the primary target for analgesia of endogenous opioid peptides, alkaloids, synthetic small molecules with diverse scaffolds, and peptidomimetics. Peptide-based opioids are viewed as potential analgesics with reduced side effects and have received constant scientific interest over the years. This study focuses on three potent peptide and peptidomimetic MOR agonists, DALDA, [Dmt^1^]DALDA, and KGOP01, and the prototypical peptide MOR agonist DAMGO. We present the first molecular modeling study and structure–activity relationships aided by in vitro assays and molecular docking of the opioid peptide analogues, in order to gain insight into their mode of binding to the MOR. In vitro binding and functional assays revealed the same rank order with KGOP01 > [Dmt^1^]DALDA > DAMGO > DALDA for both binding and MOR activation. Using molecular docking at the MOR and three-dimensional interaction pattern analysis, we have rationalized the experimental outcomes and highlighted key amino acid residues responsible for agonist binding to the MOR. The Dmt (2′,6′-dimethyl-L-Tyr) moiety of [Dmt^1^]DALDA and KGOP01 was found to represent the driving force for their high potency and agonist activity at the MOR. These findings contribute to a deeper understanding of MOR function and flexible peptide ligand–MOR interactions, that are of significant relevance for the future design of opioid peptide-based analgesics.

## 1. Introduction

Opioids are the mainstay in the management of moderate to severe pain, and remain the most efficacious analgesics currently available [1]. The opioid receptors, mu (MOR), delta (DOR), and kappa (KOR), are G protein-coupled receptors (GPCRs) and molecular targets for opioid analgesics [2], that modulate nociception pathways in the central and peripheral nervous systems (CNS and PNS) [2,3,4]. Over the years, the MOR received a constant attention as the most important opioid receptor subtype responsible for opioid-induced analgesia, but concomitantly is also most responsible for the unwanted side effects (e.g., respiratory depression, constipation, sedation, dependence, and tolerance) of opioid analgesics [1,2]. All major clinically used opioid drugs, including morphine, oxycodone, and fentanyl, are agonists at the MOR [1,5]. In the past decade, abuse and misuse of opioids became a significant public health concern due to the huge rise in overdose morbidity and mortality [6,7]. In this view, the development of effective and safer analgesics represents a key research goal for 21st century analgesic drug discovery and pain medicine.

MOR mediates not only the analgesic effect of morphine, structurally related compounds, and other opioid drugs, but it is also the endogenous target of naturally occurring peptides [3,4]. Under physiological conditions, the MOR is activated by β-endorphins, enkephalins, endomorphins, and dermorphins, as endogenous neurotransmitters that have been extensively studied since their discovery [8,9,10]. Although there is a strong evidence for their role in pain regulation and potential use as analgesics, their poor enzymatic stability and difficulties in penetrating the blood–brain barrier (BBB) after systemic administration have limited their clinical applicability [8,9,10,11,12,13,14]. Generation of potent, stable peptidomimetics with improved pharmacodynamics and pharmacokinetics entails a systematic understanding of the structure-activity relationships (SAR), where the function of key residues can be determined using different strategies, such as amino acid substitution, deletion or addition of natural or unnatural amino acids, conformational restriction through peptide main chain or side chain cyclization, peptide bond replacement, or design of bi- or multifunctional peptide ligands [11,12,13,14,15,16,17]. A diversity of opioid peptide-based analgesics with reduced adverse effects was made available through chemical synthesis and appraised as prospective therapeutic agents or research tools [5,11,13,14,15,17].

**Figure 1:**
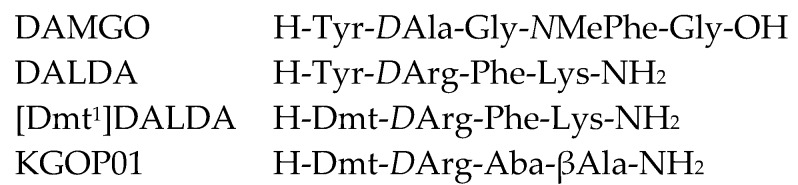
Sequence of investigated opioid peptide analogues. Dmt: 2′,6′-dimethyl-L-tyrosine; Aba: 4-amino-tetrahydro-2-benzazepinone.

Since the breakthrough of GPCR crystallization one decade ago, the understanding of the complex biology of GPCR activation and signaling has dramatically increased [16,17,18,19]. Substantial advances in structural biology of GPCRs were possible by means of innovative methodological and powerful computational systems [20,21,22]. Due to its therapeutic relevance, the MOR is among the few GPCRs determined in different activation states, with the first X-ray crystal structure of the murine MOR published in 2012 in complex with the irreversible morphinan antagonist β-funaltrexamine (PDB ID: 4DKL) [23], and the 3D-structure in the active conformation reported in 2015, where the receptor was co-crystallized with the morphinan agonist BU72 (PDB ID: 5C1M) [24]. Recently, the high resolution cryo-electron microscopy (cryo-EM) structure of the MOR (PDB ID: 6DDF) bound to the agonist peptide DAMGO (Figure 1) was reported [25], offering an important view on the structural features that contribute to the G_i_ protein-coupling specificity of the MOR. The available crystal structures of the MOR together with efficient computational methods (i.e., molecular docking and molecular dynamics simulations) provide essential insights into binding modes of ligands to the receptor, with the gained knowledge being successfully translated into the discovery of novel bioactive molecules [22,26,27]. Most of molecular modeling reports on the active and inactive structures of the MOR targeted small molecules as ligands, with only few studies employing peptides, mostly DAMGO, as the prototypical MOR selective synthetic analogue of the natural peptides enkephalin [25,28,29], endomorphin-2, and dermorphin, as endogenous opioid ligands for the MOR [30,31].

In this report, we have addressed for the first time a structure-based docking study at the active conformation of the MOR of three peptide and peptidomimetic, potent MOR agonists, DALDA, [Dmt^1^]DALDA, and KGOP01 (Figure 1). Merging experimental (in vitro assays) with computational (in silico methods) approaches, we aimed to explain the molecular basis for their binding to the MOR, in terms of understanding the structural correlations as well as interpreting the related SARs. The two peptides DALDA [32] and [Dmt^1^]DALDA [33] are synthetic analogues of the naturally-occurring dermorphin, having high enzymatic stability due to the presence of D-Arg in the second position of the peptide sequence (instead of D-Ala in dermorphin), and a modified Tyr^1^, Dmt (2′,6′-dimethyl-L-Tyr), in [Dmt^1^]DALDA (Figure 1). While DALDA does not cross the BBB to a significant extent, [Dmt^1^]DALDA was demonstrated to be able to pass the BBB to produce analgesia in animals after systemic administration [13]. KGOP01 is a new tetrapeptide, CNS penetrant, and stable analogue of [Dmt^1^]DALDA with two unnatural amino acids, 4-amino-tetrahydro-2-benzazepinone (Aba) at position 3 and βAla at position 4 [34]. The rationale for the selection of these peptide analogues is based on the numerous in vitro and in vivo studies that have established them as stable, potent MOR agonists and effective analgesics in animal pain models with an interesting pharmacology, as well as based on their value as leads in the development of new peptide ligands [13,34,35,36,37,38,39,40,41,42,43]. However, binding behavior of DALDA, [Dmt^1^]DALDA, and KGOP01 to their primary target, the MOR, using computational approaches has not been investigated up to now. The findings of this study provide structural insights into flexible peptide ligand–MOR interactions that are of significant relevance for further understanding MOR function and pharmacology, and the future design of new generation analgesics.

## 2. Results and Discussions

### 2.1. Comparison of In Vitro Binding and Activation Profiles of DALDA, [Dmt^1^]DALDA, and KGOP01 to the MOR

**Figure 2:**
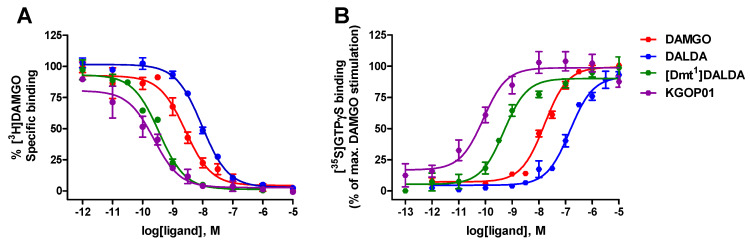
In vitro activity profiles of DAMGO, DALDA, [Dmt^1^]DALDA, and KGOP01 to the human MOR (hMOR). (**A**) Binding of tested peptides to the MOR was determined in competitive radioligand binding assays using CHO-hMOR cell membranes. (**B**) Stimulation of [^35^S]GTPγS binding by tested peptides was determined in the [^35^S]GTPγS binding assay using CHO-hMOR cell membranes. Values are expressed as the mean ± SEM (n = 3–4 independent experiments).

We have initially performed a direct comparison of in vitro activity profiles of targeted opioid peptide analogues, DALDA, [Dmt^1^]DALDA, and KGOP01 (Figure 1) at the human MOR, in terms of receptor binding and activation. For comparison purposes, the opioid binding profile of DAMGO [44], as the standard MOR agonist, is also presented. Whereas specific binding of DALDA and [Dmt^1^]DALDA to the MOR in the rat brain has been reported previously [32], with both ligands showing high affinity and selectivity for the MOR, in the present study the first data on binding affinity to the human MOR is reported. Binding to the human MOR was evaluated using in vitro competitive radioligand binding assays with membrane preparations from Chinese hamster ovary cells stably expressing the human MOR (CHO-hMOR cells) and the specific MOR radioligand [^3^H]DAMGO, according to the published procedures [43]. All three peptides displayed high capability to inhibit [^3^H]DAMGO binding to the human MOR in a concentration-dependent manner (Figure 2A), with binding affinities (as K_i_ values) in the low nanomolar to subnanomolar range (Table 1).

The high binding affinities to the human recombinant MOR expressed in CHO cells showed by DALDA and [Dmt^1^]DALDA confirms earlier data at the rat MOR in the brain tissue (K_i_ values of 1.69 nM for DALDA, and 0.143 nM for [Dmt^1^]DALDA) [32]. As shown in Table 1, replacement of the Tyr^1^ residue in DALDA with Dmt^1^ in [Dmt^1^]DALDA led to a significant increase (27-fold) in binding affinity to the human MOR, an observation that is in good agreement with findings at the rat MOR [32]. Additionally, exchanging Phe^3^-Lys^4^ residues in [Dmt^1^]DALDA with an unnatural, uncommon amino acid, respectively, in the Aba^3^-βAla^4^ sequence lead to in a new analogue, KGOP01 [34], which exhibited a further increase (ca. 2-fold) in the MOR affinity than [Dmt^1^]DALDA, and a 13-fold better MOR affinity than DAMGO (Table 1, Figure 2A).

Next, we have compared in vitro functional activities of DALDA, [Dmt^1^]DALDA and KGOP01 at the human MOR in the guanosine-5′-O-(3-[^35^S]thio)-triphosphate ([^35^S]GTPγS) binding assay using membranes from CHO cells stably expressing the human MOR, performed as described [43]. All tested peptides produced a concentration-dependent increase in the [^35^S]GTPγS binding with different levels of potencies (Figure 2B). Whereas DALDA and [Dmt^1^]DALDA showed full efficacy at the MOR, [Dmt^1^]DALDA had a considerable increased (292-fold) in agonist potency than DALDA in inducing MOR-mediated G protein activation (Table 1). Additionally, [Dmt^1^]DALDA had higher agonist potency (35-fold) than that of DAMGO. Previous in vitro bioassays using guinea-pig ileum (GPI) preparations established [Dmt^1^]DALDA as a more potent MOR agonist (180-fold) than DALDA [32]. Further, an enhanced MOR agonist potency by 5-fold was measured in the present study for KGOP01 as compared to [Dmt^1^]DALDA in the [^35^S]GTPγS binding assay (Table 1). The potent MOR agonist profile of KGOP01 was established previously in the GPI bioassay (IC_50_ = 0.8 nM) [34] and cAMP accumulation assay with HEK293 cells expressing the human MOR (EC_50_ = 0.204 nM) [42]. The outcomes derived from functional assays correlate well with the results obtained in binding studies at the MOR and structural features of investigated peptide analogues, where [Dmt^1^]DALDA and KGOP01 show a better in vitro profile than DALDA, with KGOP01 being the most potent MOR agonist of the series.

### 2.2. In Silico Investigation of DALDA, [Dmt^1^]DALDA, and KGOP01 Binding to the MOR

The observed differences in the in vitro activity profiles of DALDA, [Dmt^1^]DALDA, and KGOP01 (Table 1) encouraged in silico investigations of their binding modes at the MOR. The recently published crystal structure of the active conformation of the MOR (PDB ID: 5C1M; resolution: 2.1 Å) [24] provides the structural basis for understanding important aspects of MOR pharmacology and its function [22,24,45]. In order to examine possible binding conformations of the targeted peptide analogues to the MOR, docking experiments were performed using GOLD [46], and LigandScout [47] was used to analyze differences in receptor–ligand interactions. We used the numbering scheme from the PDB together with Ballosteros–Weinstein nomenclature.

**Figure 3:**
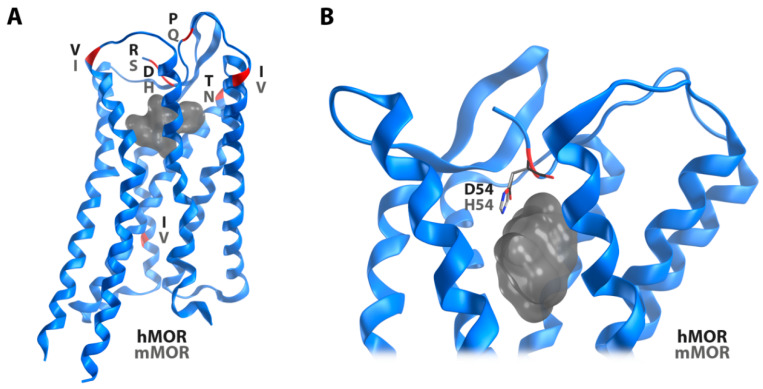
The murine (PDB ID: 5C1M) and the human MOR model differ only in seven amino acid residues as illustrated in red (**A**). Only one of these differing residues is directly pointing to the ligand binding site (**B**, grey surface). Whereas a histidine residue is at position 54 in the murine MOR, the human receptor has an aspartic acid at this position.

**Figure 4:**
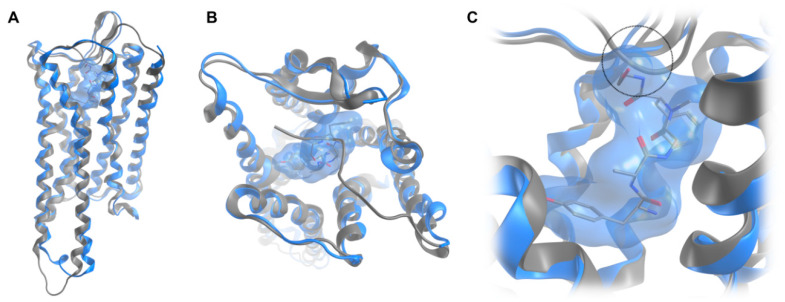
Superimposition of the crystal structure of the MOR (PDB ID: 5C1M) with co-crystallized BU72 (grey), and the recently available cryo-EM structure (PDB ID: 6DDF) with bound DAMGO (blue) in a transmembrane (**A**) and extracellular view (**B**). The close-up view on DAMGO (blue surface) in the binding site (**C**) unveils a sterical clash (circle) of the peptide with the N-terminus resolved in the active crystal structure.

Since the available crystal structure of the active MOR (PDB ID: 5C1M) [24] represents the murine receptor, a structural model of the human MOR was built by in silico mutations of differing residues. Interestingly, six out of seven differing amino acid residues are located in the extracellular region. The high similarity in the receptor core region and the intracellular side suggests a conserved receptor activation mechanism, but potential differences for ligand recognition (Figure 3). However, only one of these residues turned out to directly point to the ligand binding site. Instead of a histidine at position 54 in the murine MOR, the human receptor has an aspartic acid at this position. Notable, the recently reported cryo-EM structure of the MOR (PDB ID: 6DDF; resolution: 3.5 Å) [25] bound to the agonist peptide DAMGO misses the *N*-terminal region and unveils a binding mode for DAMGO, which is not compatible with the previous crystal structure [24] (Figure 4), indicating that DAMGO might bind differently in the truncated vs. untruncated receptor. Due to the fact that the cryo-EM structure presents the MOR bound to DAMGO, this structure was subsequently used for binding mode investigations of DALDA, [Dmt^1^]DALDA, and KGOP01, with the same in silico mutations as in the abovementioned structural model of the human MOR. The discrepancies between the crystal structure and the cryo-EM structure with regard to the *N*-terminus suggests an important, but different role in binding of non-peptide ligands, such as morphinan-based agonists and peptide ligands. We would like to note that binding mode predictions are always of hypothetic nature and in this specific case the reliability of our proposed interaction pattern strongly depends on the receptor region. Whereas the *C*-terminal parts of the studied peptides, located in the receptor core region are more reliable, the missing structural information for the *N*-terminus of the receptor makes the binding orientations of the *N*-terminal parts of the studied peptides and resulting interactions more speculative. 

**Figure 5:**
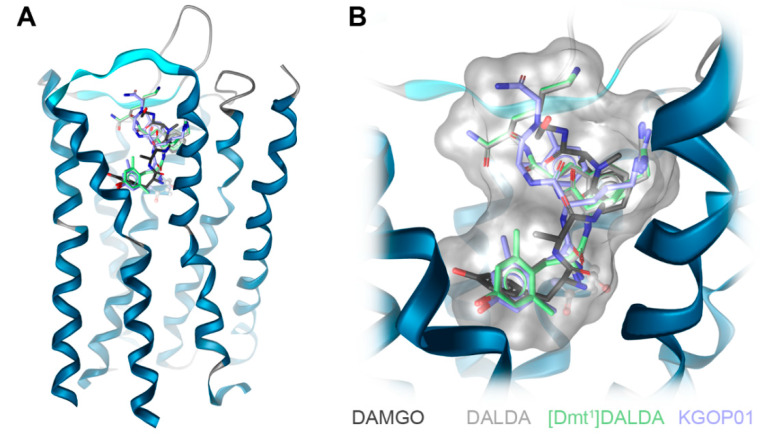
Investigated opioid peptides DAMGO, DALDA, [Dmt^1^]DALDA, and KGOP01 show a comparable binding orientation in the inner core region of the human MOR model (based on PDB ID: 6DDF) (**A**). Aspartic acid D147^3.32^ plays a major role in ligand binding through a charge interaction with the opioid peptides (**B**).

**Figure 6:**
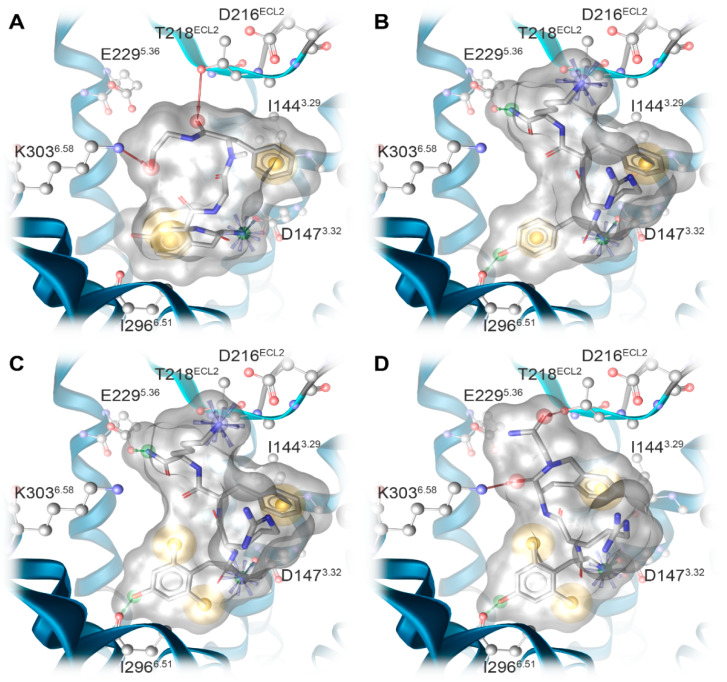
Predicted binding modes at the human MOR model (based on PDB ID: 6DDF) and receptor–ligand interaction patterns of opioid peptides (**A**) DAMGO, (**B**) DALDA, (**C**) [Dmt^1^]DALDA, and (**D**) KGOP01. Yellow spheres indicate lipophilic contacts, red arrows hydrogen bond acceptors, green arrows hydrogen bond donors, and positively charged centers are shown as blue spheres.

**Figure 7:**
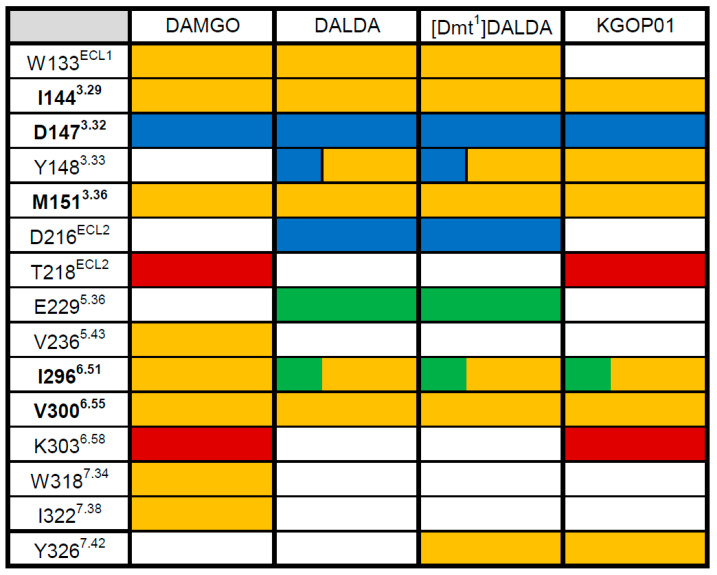
Ligand–MOR interaction pattern derived from molecular docking solutions of opioid peptides DAMGO, DALDA, [Dmt^1^]DALDA, and KGOP01. Yellow fields indicate lipophilic contacts, red fields hydrogen bond acceptors, green fields hydrogen bond donors, and positively charged centers are shown as blue fields. White fields indicate the absence of an interaction with that residue. Residues which show the same type of interaction for all morphinan ligands are marked in bold.

**Figure 8:**
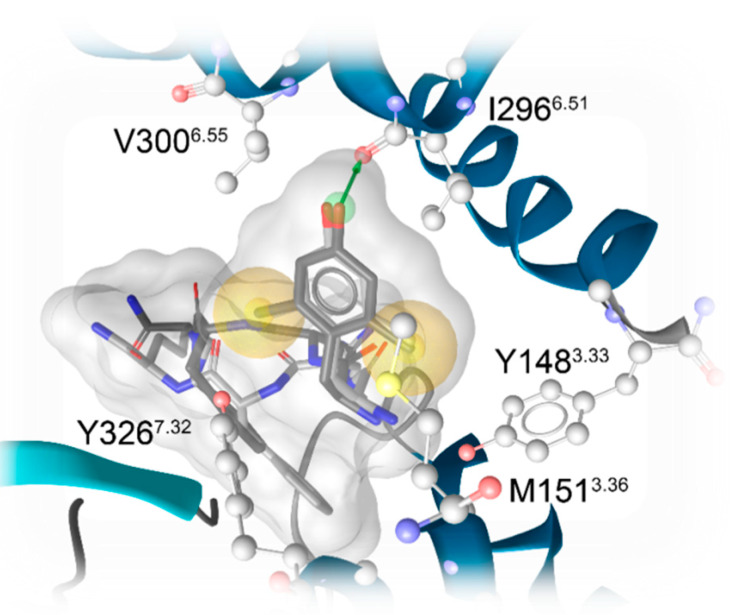
Hydrophobic environment of Dmt moieties of [Dmt^1^]DALDA (light grey) and KGOP01 (dark grey). The two methyl groups show additional hydrophobic contacts with Y148^3.33^ and Y326^7.32^, respectively, which are not observable for DAMGO or DALDA. Yellow spheres indicate lipophilic contacts and green arrows hydrogen bond donors. For clarity, only interactions of the Dmt moiety are shown.

Docking of DAMGO, DALDA, [Dmt^1^]DALDA, and KGOP01 to the structural model of the human MOR (PDB ID: 6DDF) resulted in comparable binding orientations for the four peptides (Figure 5). Several receptor–ligand interactions were observed in all complexes (Figure 6), and an overview of detected receptor–ligand interactions is presented in Figure 7. Due to missing information on the role of the receptor’s *N*-terminus for ligand binding and the high flexibility of the peptide ligands, the interactions with the extracellular loop regions are more speculative than the interactions within the inner core region. As expected, D147^3.32^ forms a charge interaction with the primary amine of the tyrosine (in DAMGO and DALDA) or the Dmt (in [Dmt^1^]DALDA and KGOP01). Additionally, this primary amine of the tyrosine of DALDA and [Dmt^1^]DALDA forms a π–cation interaction with Y148^3.33^. The central role of D147^3.32^ and Y148^3.33^ for binding of DAMGO, morphine and morphinan ligands, and other small molecules to the MOR is well-recognized [23,24,25,26,27,28,48]. The phenol moieties of all ligands are pointing towards I296^6.51^ and form a hydrogen bond except for DAMGO. In theory, the phenol moieties could also form hydrogen bonds to water molecules as observed in the MOR crystal structure (PDB ID: 5C1M), but the role of water-mediated interaction networks for peptide ligands is still not clear. The phenyl rings of the phenylalanine (in DAMGO, DALDA, and [Dmt^1^]DALDA) fill a hydrophobic pocket that comprises the aliphatic chain of I144^3.29^ residues. While the Aba moiety of KGOP01 only reaches I144^3.29^, it also makes KGOP01 more rigid and thereby allows for a potentially highly favorable hydrogen bond with K303^6.58^. Interestingly, the methyl group of the alanine of DAMGO shows unique lipophilic contacts with W318^7.34^ and I322^7.38^ residues, which could not be observed for the other peptides. In comparison, DAMGO and KGOP01 are more similar in terms of their hydrogen bonding pattern including T218^ECL2^ and K303^6.58^. Further, DALDA and [Dmt^1^]DALDA only differ in the additional lipophilic contact of [Dmt^1^]DALDA with Y326^7.42^. Overall, the four targeted opioid peptides show comparable binding modes to the MOR. The tyrosine/Dmt ring of all four peptides shows lipophilic contacts with M151^3.36^, I296^6.51^, and V300^6.55^ residues. The two methyl groups of the Dmt moiety in [Dmt^1^]DALDA and KGOP01 show additional lipophilic contacts with Y148^3.33^ and Y326^7.42^ residues, which might lower the entropic penalty upon binding (Figure 8). The latter rigidification effect might also strengthen the hydrogen bond with I296^6.51^ residue (Figure 6).

## 3. Materials and Methods

### 3.1. Chemicals and Materials

Cell culture media and supplements were obtained from Sigma-Aldrich Chemicals (St. Louis, MO, USA). Radioligands [^3^H]DAMGO (50 Ci/mmol) and [^35^S]GTPγS (1250 Ci/mmol) were purchased from PerkinElmer (Boston, MA, USA). DAMGO, unlabeled GTPγS, and guanosine diphosphate (GDP) were obtained from Sigma-Aldrich Chemicals (St. Louis, MO, USA). All other chemicals were of analytical grade and obtained from standard commercial sources.

### 3.2. Peptide and Peptidomimetic Ligands

DALDA [32], [Dmt^1^]DALDA [33], and KGOP01 [34] were synthesized as described previously [34], with purities >98%. DAMGO was obtained from Sigma-Aldrich Chemicals (St. Louis, MO, USA). Test peptides were prepared as 1 mM stocks in water, and further diluted to working concentrations in the appropriate medium.

### 3.3. Cell Culture

CHO cells stably expressing the human MOR were kindly provided by Dr. Lawrence Toll (SRI International, Menlo Park, CA, USA). The CHO-hMOR cell line was grown in Dulbecco’s Minimal Essential Medium (DMEM)/Ham’s F-12 medium supplemented with fetal bovine serum (FBS, 10%), penicillin/streptomycin (0.1%), L-glutamine (2 mM), and geneticin (400 µg/mL). Cells were maintained at 37 °C in 5% CO_2_ humidified air.

### 3.4. Competitive Radioligand Binding Assays

Binding assays were conducted on human MOR stably transfected into CHO cells (CHO-hMOR) according to the published procedure [43]. Cell membranes were prepared as described previously, and stored at −80 °C until use [43]. Protein concentration of cell membrane preparations was determined by the method of Bradford using bovine serum albumin as the standard [49]. Cell membranes (15–20 µg) were incubated in 50 mM Tris-HCl buffer (pH 7.4) with [^3^H]DAMGO (1 nM) and various concentrations of test peptides in a final volume of 1 mL, for 60 min at 25 °C. Non-specific binding was determined using 10 µM of unlabeled DAMGO. After incubation, reactions were terminated by rapid filtration through Whatman glass GF/C fiber filters. Filters were washed three times with 5 mL of ice-cold 50 mM Tris-HCl buffer (pH 7.4) using a Brandel M24R cell harvester (Gaithersburg, MD, USA). Radioactivity retained on the filters was counted by liquid scintillation counting using a Beckman Coulter LS6500 (Beckman Coulter Inc., Fullerton, CA, USA). The inhibitory constant (K_i_, in nM) values were calculated from the competition binding curves by nonlinear regression analysis and the Cheng–Prusoff equation [50]. All experiments were performed in duplicate and repeated at least three times. 

### 3.5. [^35^S]GTPγS Binding Assays

Binding of [^35^S]GTPγS to membranes from CHO cells stably expressing the human MOR(CHO-hMOR) was conducted according to the published procedure [43]. Cell membranes (5-10 µg) in 20 mM HEPES, 10 mM MgCl_2_, and 100 mM NaCl, pH 7.4 were incubated with 0.05 nM [^35^S]GTPγS, 10 µM GDP and various concentrations of test peptides in a final volume of 1 mL, for 60 min at 25°C. Non-specific binding was determined using 10 µM GTPγS, and the basal binding was determined in the absence of test ligand. Samples are filtered over glass Whatman glass GF/B fiber filters and counted as described for binding assays. In each individual experiment, the increase in [^35^S]GTPγS binding produced by the test peptides were normalized to the maximal stimulation of the reference full MOR agonist, DAMGO and nonlinear regression performed on each individual curve were averaged to yield potency (EC_50_, in nM) and efficacy (as % stim.) values. All experiments were performed in duplicate and repeated at least three times.

### 3.6. Data Analysis

Experimental data were analyzed and graphically processed using the GraphPad Prism 5.0 Software (GraphPad Prism Software Inc., San Diego, CA, USA), and are presented as means ± SEM.

### 3.7. Molecular Modeling

The structure of the human MOR was remodeled based on the crystal structure of the murine MOR (PDB ID: 5C1M) [24] by using the mutation tool of Molecular Operating Environment (MOE, 2019.0101; Chemical Computing Group Inc., Montreal, QC, Canada) with subsequent sidechain optimization. Complementary, the cryo-EM structure of the MOR with bound DAMGO (PDB ID: 6DDF) [25] was used. All receptor-ligand docking experiments were performed with the CCDCs software GOLD version 5.7.0 [46]. Water molecules and ligands were removed. Assignment of protonation states and protein preparation were performed using Protonate3D [51] (implemented in MOE 2019.1, Chemical Computing Group, Montreal, QC, Canada). All residues of the inner receptor core region and the C-terminal domain were defined as potential binding site (12 Å around the γ-carbon atom of D147; PDB ID: 6DDF) [25]. For receptor-ligand docking, default settings were applied and GoldScore served as primary scoring function with DAMGO as reference ligand. All obtained docking poses and receptor–ligand interactions were analyzed using LigandScout 4.4 [47] using a 3D-pharmacophore approach [52]. 

## 4. Conclusions

Given the essential clinical role of the MOR in mediating pain inhibition and other physiological activities, with endogenous peptides as natural agonists of the MOR, a basic understanding of the binding mechanism of opioid peptides to the MOR is required for their further development as potential analgesics and drugs for pain treatment and other human disorders. The peptidic nature of endogenous MOR agonists provides a variety of modification possibilities to design specific and stable MOR agonists. In this study, we have reported on a set of peptide analogues, DAMGO, DALDA, [Dmt^1^]DALDA, and KGOP01, for which in silico binding modes and in vitro activities at the MOR were correlated. The present results evidence the consequence of the modified Tyr^1^, Dmt, in [Dmt^1^]DALDA and KGOP01 on the pharmacological profile with molecular docking studies offering a structural basis for the observed MOR activities. In vitro receptor binding and functional assays revealed the same rank order with KGOP01 > [Dmt^1^]DALDA > DAMGO > DALDA for both binding and MOR activation. In silico binding mode investigations indicated the important contribution of the Dmt moiety for binding and MOR activation, specifically, with the two methyl groups of the Dmt moiety in [Dmt^1^]DALDA and KGOP01 showing additional lipophilic contacts with Y148^3.33^ and Y326^7.42^ residues. Generally, the limited CNS penetration of peptides often impairs their development as therapeutics. Furthermore, the feasibility of peptides for clinical application is much precluded by their enzymatic degradation. DALDA, [Dmt^1^]DALDA, and KGOP01 have high stability against enzymatic degradation, due to the presence of certain structural modifications, i.e., unnatural and synthetic amino acids. While DALDA does not cross the BBB, the [Dmt^1^]DALDA and KGOP01 can enter the CNS [13,42]. The gained knowledge from this study on which molecular interactions with the MOR these opioid peptides share and distinguish them, with Y148^3.33^ and Y326^7.42^ sites being of significance, may also help to understand the differences in the pharmacokinetics between these peptides. Our findings offer structural insights into flexible peptide ligand–MOR interactions that are important for further understanding of MOR function and pharmacology, and the future design of peptide-based analgesics.

## Figures and Tables

**Table 1:** In vitro binding and agonist activity of opioid peptide analogues at the human MOR.

Opioid Peptide	Binding Affinity ^a^	Agonist Activity ^b^	
	K_i_ (nM)	EC_50_ (nM)	% stim.
DAMGO	1.46 ± 0.37	18.1 ± 2.0	100
DALDA	6.36 ± 0.24	149 ± 28	92 ± 2
[Dmt^1^]DALDA	0.23 ± 0.02	0.51 ± 0.06	90 ± 4
KGOP01	0.11 ± 0.05	0.10 ± 0.02	99 ± 6

^a^ Determined in competitive radioligand binding assays using membrane from CHO expressing the human MOR (CHO-hMOR). ^b^ Determined in the [^35^S]GTPγS binding assay using CHO-hMOR cell membranes. Percentage stimulation (% stim.) relative to DAMGO (reference MOR full agonist). Values are means ± SEM (n = 3-4 independent experiments).
